# Targeting type-2 metabotropic glutamate receptors to protect vulnerable hippocampal neurons against ischemic damage

**DOI:** 10.1186/s13041-015-0158-2

**Published:** 2015-10-24

**Authors:** Marta Motolese, Federica Mastroiacovo, Milena Cannella, Domenico Bucci, Anderson Gaglione, Barbara Riozzi, Robert Lütjens, Sonia M. Poli, Sylvain Celanire, Valeria Bruno, Giuseppe Battaglia, Ferdinando Nicoletti

**Affiliations:** Istituto di Ricovero e Cura a Carattere Scientifico (IRCCS) Neuromed, 86077 Pozzilli, Italy; Addex Therapeutics, Plan-les-Ouates, Geneva, Switzerland; Department of Physiology and Pharmacology, Sapienza University of Rome, Piazzale Aldo Moro, 5, 00185 Rome, Italy; Present address: Pragma Therapeutics, 74166 Saint-Julien-en-Genevois, France

**Keywords:** ADX92639, Epigenetics, Global ischemia, mGlu2 receptors, Neuronal vulnerability

## Abstract

**Background:**

To examine whether metabotropic glutamate (mGlu) receptors have any role in mechanisms that shape neuronal vulnerability to ischemic damage, we used the 4-vessel occlusion (4-VO) model of transient global ischemia in rats. 4-VO in rats causes a selective death of pyramidal neurons in the hippocampal CA1 region, leaving neurons of the CA3 region relatively spared. We wondered whether changes in the expression of individual mGlu receptor subtypes selectively occur in the vulnerable CA1 region during the development of ischemic damage, and whether post-ischemic treatment with drugs targeting the selected receptor(s) affords neuroprotection.

**Results:**

We found that 4-VO caused significantly reduction in the transcript of mGlu2 receptors in the CA1 region at times that preceded the anatomical evidence of neuronal death. Down-regulation of mGlu2 receptors was associated with reduced H3 histone acetylation at the Grm2 promoter. The transcripts of other mGlu receptor subtypes were unchanged in the CA1 region of 4-VO rats. Ischemia did not cause changes in mGlu2 receptor mRNA levels in the resistant CA3 region, which, interestingly, were lower than in the CA1 region. Targeting the mGlu2 receptors with selective pharmacologic ligands had profound effects on ishemic neuronal damage. Post-ischemic oral treatment with the selective mGlu2 receptor NAM (negative allosteric modulator), ADX92639 (30 mg/kg), was highly protective against ischemic neuronal death. In contrast, s.c. administration of the mGlu2 receptor enhancer, LY487379 (30 mg/kg), amplified neuronal damage in the CA1 region and extended the damage to the CA3 region.

**Conclusion:**

These findings suggest that the mGlu2 receptor is an important player in mechanisms regulating neuronal vulnerability to ischemic damage, and that mGlu2 receptor NAMs are potential candidates in the experimental treatments of disorders characterized by brain hypoperfusion, such as hypovolemic shock and cardiac arrest.

## Introduction

Understanding the mechanisms underlying neuronal vulnerability to ischemic damage may pave the way to novel therapeutic strategies in cerebrovascular disorders. Transient cerebral global ischemia in experimental animal models or humans causes the degeneration of specific populations of vulnerable neurons, such as pyramidal neurons of the CA1 hippocampal subfield [1, 2]. Neurodegeneration develops slowly and becomes manifest only several hours following reperfusion [2]. This relatively long temporal window facilitates the study of the molecular mechanisms underlying the delayed ischemic neuronal death, and allows the translation of these mechanisms into potential therapeutic targets. One of the most widely used animal models of transient global ischemia is the 4-vessel occlusion (4-VO) model in rats [3]. In rats subjected to 4-VO, CA1 pyramidal neurons tipically die at 48-72 h after reperfusion, whereas neurons of the CA3 region and the dentate gyrus are relatively spared, at least at this timepoint [4, 5]. The use of this model led to the identification of early molecular events occurring in neurons destined to die, such as derepression of the gene silencer, REST [6], down-regulation of the Ca^2+^-impermeable AMPA receptor subunit, GluA2 [2, 4, 7], activation of nuclear factor-κΒ (NFκΒ) and cyclooxygenase-2 [8, 9], and induction of the Wnt inhibitor, Dickkopf-1 [5]. These findings laid the groundwork for pharmacological studies showing that AMPA receptor antagonists are able to protect hippocampal CA1 neurons against ischemic damage [10–12].

Subtype-selective ligands of metabotropic glutamate (mGlu) receptors have shown efficacy as neuroprotective drugs in models of transient global ischemia [13]. Pellegrini-Giampietro and collegues have consistently shown that mGlu1, but not mGlu5, receptor antagonists are protective against ischemic damage of CA1 pyramidal neurons in gerbils exposed to global ischemia and in organotypic hippocampal slices exposed to oxygen/glucose deprivation [14, 15, 16]. Expression and function of mGlu5 receptors are decreased in the hippocampal CA1 region at early times after transient global ischemia [17–19], whereas mGlu1 mRNA levels are either decreased [17] or unchanged [18].

Mixed orthosteric agonists of mGlu2 and mGlu3 receptors (compounds LY354740 and LY379268) showed also neuroprotective activity in the gerbil model of transient global ischemia [20, 21], raising the important question of which of the two subtypes should be specifically targeted by therapeutic interventions. Interestingly, studies carried out in cultured neurons suggest that activation of mGlu3 receptors is neuroprotective, whereas activation of mGlu2 receptors is not harmful under control conditions, but amplifies neuronal damage if combined with neurotoxic insults, such as N-methyl-D-aspartate (NMDA) [22] or β-amyloid peptide [23]. *In situ* hybridization analysis in the hippocampus of rats subjected to transient global ischemia showed either increases [18] or reductions [17] in mGlu2 receptor mRNA at 24 h after reperfusion, with no changes in the transcript of mGlu3 receptors. Whether expression of mGlu2 and mGlu3 receptors differs in vulnerable and resistant hippocampal subregions in response to global ischemia is unknown.

We now report that transient global ischemia causes an epigenetic down-regulation of mGlu2 receptors, which selectively occurs in the vulnerable CA1 region. Post-ischemic treatment with a selective negative allosteric modulator (NAM) of mGlu2 receptors protected CA1 neurons against ischemic damage, whereas treatment with a positive allosteric modulator (PAM) of mGlu2 receptors extended ischemic damage to CA3 neurons.

## Results

### Temporal profile of neuronal damage in CA1 and CA3 regions in rats subjected to transient global brain ischemia

**Fig. 1:**
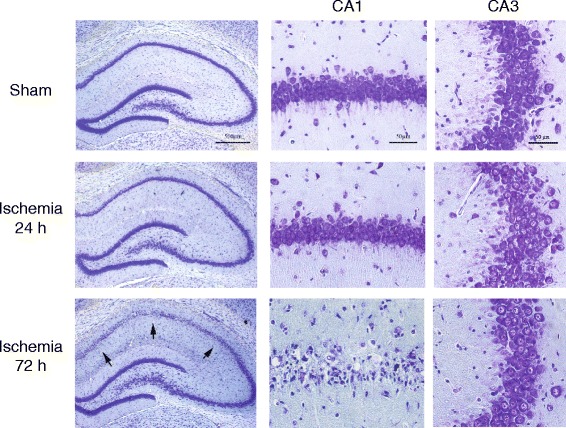
Temporal profile of neuronal damage in CA1 and CA3 hippocampal regions. Representative Nissl staining of the CA1 and CA3 regions of rats subjected to 4-VO at different times after reperfusion (24 and 72 h)

Transient global ischemia induced by 4-VO in rats caused the expected loss of CA1 pyramidal neurons at 72 h following reperfusion, with no detectable neuronal death at 6, 12, or 24 h (images at 24 and 72 h are shown in Fig. 1). Neurons of the CA3 region and the dentate gyrus were largely resistant to ischemic damage, at least at 72 h after reperfusion (Fig. 1).

### Global ischemia caused an early down-regulation of mGlu2 receptors in the CA1 region

**Fig. 2:**
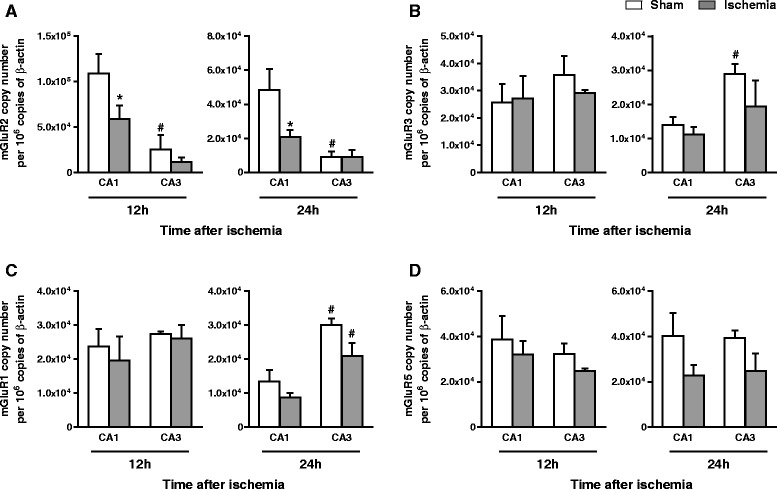
Expression profile of mGlu receptors in CA1 and CA3 regions at times preceding neuronal death. Quantitative PCR analysis of (**a**) mGlu2, (**b**) mGlu3, (**c**) mGlu1 and (**d**) mGlu5 receptors (mGluR1, -2, -3, and -5) at 12 and 24 h after reperfusion. mRNA values, expressed as copy number, were normalized to β-actin and are means ± S.E.M. of 3-5 rats per group; One-way ANOVA + Fisher's LSD: In (**a**) mGluR2 at 12 h post ischemia: F_(3,15)_ = 7.6, *p* < 0.05; mGluR2 at 24 h post ischemia: F_(3,15)_ = 7.1, *p* < 0.05; in (**b**) mGluR3 at 24 h: F_(3,14)_ = 2.6, *p* < 0.05; in (**c**) mGluR1 at 24 h post ischemia: F_(3,14)_ = 9.2, *p* < 0.05. Post-hoc analysis: *p* < 0.05 vs. the respective values obtained in sham operated rats (*); or vs. the respective values obtained in the CA1 region (#)

We measured the transcripts of mGlu1, mGlu2, mGlu3, and mGlu5 receptors by qPCR in microdissected CA1 and CA3 regions of sham-operated and 4-VO rats at times that preceded neuronal death (12 and 24 h after reperfusion). We were surprised to find that sham-operated rats showed much lower mGlu2 mRNA levels in CA3 than in CA1 region at both times (Fig. 2a). This distribution pattern was unique to mGlu2 receptors, at least with respect to mGlu1, mGlu3, and mGlu5 receptors (Fig. 2b-d). 4-VO Rats showed a substantial decrease in mGlu2 mRNA levels in the CA1 region, with no changes in the CA3 region. This reduction was more prominent at 24 h than at 12 h after reperfusion (-57 % and -46 % vs. the respective values of sham-operated rats, respectively) (Fig. 2a). Ischemia did not cause significant changes in mGlu1, mGlu3, and mGlu5 receptor mRNA levels in the two hippocampal subregions (Fig. 2b-d). At 24 h, mGlu1 mRNA levels were higher in CA3 than in CA1 region in both sham-operated and ischemic rats (Fig. 2c).

### Global ischemia up-regulated HDAC2 and reduced histone acetylation in the mGlu2 receptor gene promoter in the CA1 region at times that precede neuronal death

**Fig. 3:**
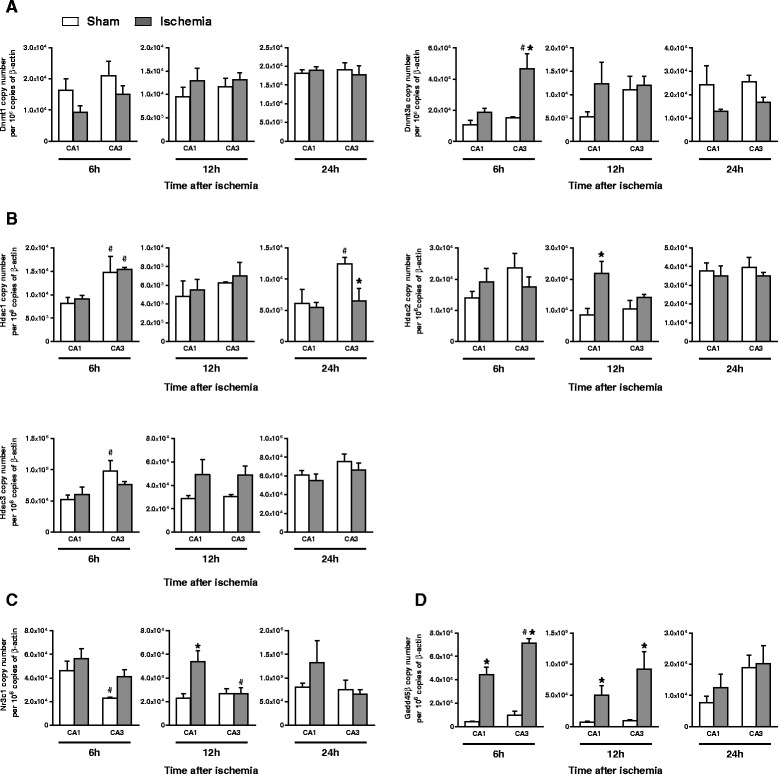
Gene expression analysis of epigenetic factors following global ischemia at several times preceding neuronal death. Quantitative PCR analysis of (**a**) Dnmt1 and Dnmt3a, (**b**) Hdac1, Hdac2 and Hdac3, (**c**) Nr3c1 and (**d**) Gadd45β, at 6, 12 and 24 h after reperfusion. mRNA values, expressed as copy number, were normalized to β-actin and are means ± S.E.M. of 3-5 individual determinations; One-way ANOVA + Fisher's LSD. In (**a**) Dnmt3a at 6 h: F_(3,15)_ = 9.3, *p* < 0.05; In (**b**) Hdac1 at 6 h: F_(3,17)_ = 4.8, *p* < 0.05; at 24 h: F_(3,15)_ = 3.8, *p* < 0.05; Hdac2 at 12 h: F_(3,13)_ = 4.6, *p* < 0.05; Hdac3 at 6 h: F_(3,17)_ = 2.9, *p* < 0.05; in (**c**) Nr3c1 at 6 h: F_(3,17)_ = 4.3, *p* < 0.05; Nr3c1 at 12 h: F_(3,14)_ = 5.165 *p* < 0.05 and in (**d**) Gadd45β at 6 h: F_(3,17)_ = 48.4, *p* < 0.0001, at 12 h: F_(3,13)_ = 8.6 *p* < 0.005. Post-hoc analysis: *p* < 0.05 vs. the respective values obtained in sham operated rats (*); or vs. the respective values obtained in the CA1 region (#)

In order to dissect the epigenetic programming leading to changes in the expression of mGlu2 receptors in response to global ischemia, we measured the transcript encoding for type-1 and type-3a DNA-methyltransferases (Dnmt1 and -3a) and type-1, -2, and -3 histone deacetylases (Hdac1, -2 and -3) in the CA1 and CA3 regions in 4-VO rats at 6, 12, and 24 h after reperfusion and at corresponding times in sham-operated rats. We also measured the transcript of Gadd45β, which is the product of a DNA-damage responsive gene and is involved in mechanisms of DNA demethylation [24–26], and the transcript of the glucocorticoid receptor (GR, encoded by the Nr3c1 gene), which is known to regulate the expression of Hdac2 [27]. Transient global ischemia caused a selective increase in the transcripts of Hdac2 and GRs in the vulnerable CA1 region at 12 h after reperfusion (Fig. 3b, c). The transcript encoding for Gadd45β was strongly up-regulated at early times after reperfusion (6 and 12 h) in both CA1 and CA3 (Fig. 3d; see also ref. [28]), suggesting that the resistant CA3 region was also reactive to the ischemic insult at early times after reperfusion. The transcripts of Dnmt1 and Dnmt3a did not change in CA1 in response to global ischemia. Increases in Dnmt3a mRNA levels were only observed in the CA3 region at 6 h after ischemia (Fig. 3a). Hdac1 and Hdac3 mRNA levels did no change in CA1 of 4-VO rats. We only found a reduction of Hdac1 mRNA levels in CA3 at 24 h after ischemia with respect to the corresponding group of sham-operated rats (Fig. 3b).

**Fig. 4:**
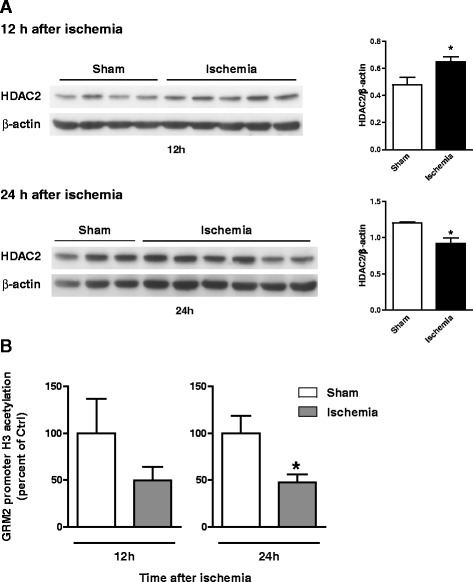
HDAC2 protein levels and Grm2 promoter H3-acetylation in the CA1 region following global ischemia. (**a**) Western blot analysis of HDAC2 in CA1 region at 12 and 24 h after reperfusion. Values are means ± S.E.M of 3-6 rats per group * *p* < 0.05 (Student's t test) vs. sham-operated rats; t_(7)_ = 2.73 and t_(7)_ = 2.61 at 12 and 24 h, respectively. (**b**) Chip analysis of H3 histone acetylation of Grm2 promoter in CA1 region at 12 and 24 h after reperfusion. Values (percent of input) are expressed as percent of controls (sham-operated), and are means ± S.E.M. of 3-8 rats per group. *p < 0.05 (Student's t-test) vs. sham-operated rats; t_(5)_ = 1.43 and t_(13)_ = 2.67 at 12 and 24 h, respectively

Knowing that the mGlu2 receptor gene (Grm2) gene promoter is targeted by HDAC2 [29], we extended the analysis to the HDAC2 protein levels and to histone H3 acetylation at the mGlu2 receptor gene promoter in the CA1 region at 12 and 24 h after reperfusion. Immunoblot analysis showed a significant increase in HDAC2 protein levels at 12 h, and a reduction at 24 h after reperfusion (Fig. 4a). ChIP analysis of acetylated-H3 histone bound to the Grm2 promoter showed a trend to a reduction (-50 %) at 12 h, and a significant reduction (-53 %) at 24 h after reperfusion (Fig. 4b).

### Effect of post-ischemic treatment with selective mGlu2 receptor ligands on neuronal damage in 4-VO rats

Because orthosteric ligands do not differentiate between mGlu2 and mGlu3 receptors, we used two brain permeant allosteric modulators to specifically examine the influence of mGlu2 receptors on neurodegeneration/neuroprotection in the 4-VO model of transient global ischemia. As a selective mGlu2 PAM, we used a commercially available compound (LY487379), which has no intrinsic agonist activity at mGlu2 receptors, but markedly potentiates glutamate-evoked responses at mGlu2 receptors [30, 31]. To selectively inhibit mGlu2 receptors, we used compound ADX92639, the characterization of which is reported below.

### *In vitro* pharmacological profile of ADX92639

**Fig. 5:**
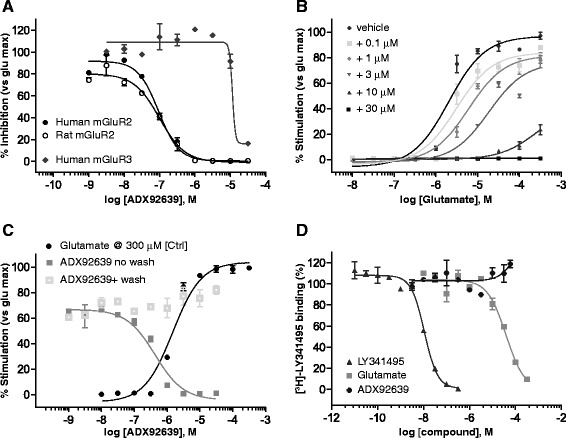
*In vitro* pharmacological profile of the mGlu2 receptor NAM ADX92639. FLIPR data are shown in (**a-c**). Concentration-response curves of ADX92639 in HEK293 cells stably expressing human or rat mGlu2 receptors or human mGlu3 receptor and challenged with glutamate at the EC_80_ value are shown in (**a**). Schild-plot analysis carried out in human mGlu2 cells challenged with glutamate in the absence or presence of increasing concentrations of ADX92639 is shown in (**b**). A reversibility experiment is shown in (**c**), where human mGlu2 receptor cells were treated with increasing concentrations of ADX92639 before being either washed or not prior to the addition of an EC_80_ of glutamate. [^3^H]LY341495 (1 nM) binding in membrane prepared from human mGlu2 clones incubated in the absence or presence of increasing concentrations of ADX92639, glutamate or non-radioactive LY341495 is shown in (**d**). Values are means ± SD of two replicates per point and curves are representative of one from 2 to 6 separate experiments

Compound ADX92639 was tested in an agonist or antagonist mode with a FLIPR-based intracellular Ca^2+^ mobilization assay using HEK293 cells stably expressing rat or human recombinant mGlu receptor subtypes. ADX92639 exhibited a high potency as an mGlu2 receptor antagonist, with apparent IC_50_ values of 175 nM and 145 nM at human and rat mGlu2 receptor clones, respectively (Fig. 5a). The potency as an antagonist of ADX92639 at human mGlu3 receptor clones was 100 fold lower (IC_50_ value = 17.4 μM) (Fig. 5a). The compound had no activity as an agonist, antagonist, PAM or NAM at all other mGlu receptor subtypes (data not shown). In addition, ADX92639 was inactive up to 10 μM in competition binding assay on membranes expressing 71 G-protein coupled receptors, transporters, enzymes, and ion channels (binding profile at Cerep, data not shown).

Schild-plot experiments carried out in human mGlu2 receptor clones showed that ADX92639 induced a rightward shift of the glutamate concentration-response curve (in the FLIPR assay) together with a decrease in glutamate efficacy (Fig. 5b). This profile is consistent with a NAM activity of ADX92639 at mGlu2 receptors. Reversibility experiments (Fig. 5c) showed that the effect of ADX92639 observed in the Schild-plot analysis was not due to a non-reversible competitive effect of the compound.

The non competitive nature of the antagonist activity of ADX92639 was confirmed by competition experiments using [^3^H]LY341495, in which ADX92639 was unable to displace specifically bound [^3^H]LY341495 to membranes from human mGlu2 receptor clones, as opposed to LY341495 and glutamate (Fig. 5d).

### Repeated treatment with ADX92639 did not cause changes in body temperature in rats

Knowing that mGlu2 receptor ligands may cause changes in body temperature [32–35] we measured body temperature in rats after oral administration of ADX92639 or its vehicle. ADX92639 or vehicle was administered three times with intervals of 24 h. Values of body temperature, collected at different times from 1 to 58 h after the first administration, did not differ between the groups of rats treated with ADX92639 and vehicle. In none of the timepoints values were lower than 35.8 °C in both groups of rats (Table 1).

**Table 1:** Effects of ADX92639 or its vehicle on body temperature at different times (1-58 h) after first administration

Hours	Vehicle	ADX92639
0	36.9 ± 0.3	37.0 ± 0.1
1	36.4 ± 0.3	36.5 ± 0.3
3	35.9 ± 0.1	36.1 ± 0.1
6	35.8 ± 0.1	35.8 ± 0.1
9	37.0 ± 0.1	36.9 ± 0.2
24	36.2 ± 0.1	36.2 ± 0.4
25	35.9 ± 0.0	35.8 ± 0.1
27	35.9 ± 0.0	35.9 ± 0.1
30	36.2 ± 0.2	36.3 ± 0.2
33	36.8 ± 0.3	36.1 ± 0.2
48	36.0 ± 0.1	35.9 ± 0.0
49	36.1 ± 0.0	36.1 ± 0.0
52	36.1 ± 0.1	36.1 ± 0.1
55	36.2 ± 0.0	36.2 ± 0.1
58	36.7 ± .03	36.5 ± 0.3

Effect of oral treatment with ADX92639 (30 mg/kg) or vehicle on body temperature in normal rats. ADX92639 or vehicle were administered three times with 24 h of interval. Rectal temperature was measured at different times (1-58 h) after the first administration of ADX92639 or vehicle. Value are means ± S.E.M. (*n* = 3)

### Treatment with the mGlu2 receptor negative allosteric modulator, ADX92639, protected CA1 neurons against ischemic damage, whereas treatment with the mGlu2 receptor enhancer, LY487379, extended ischemic damage to CA3 neurons

**Fig. 6:**
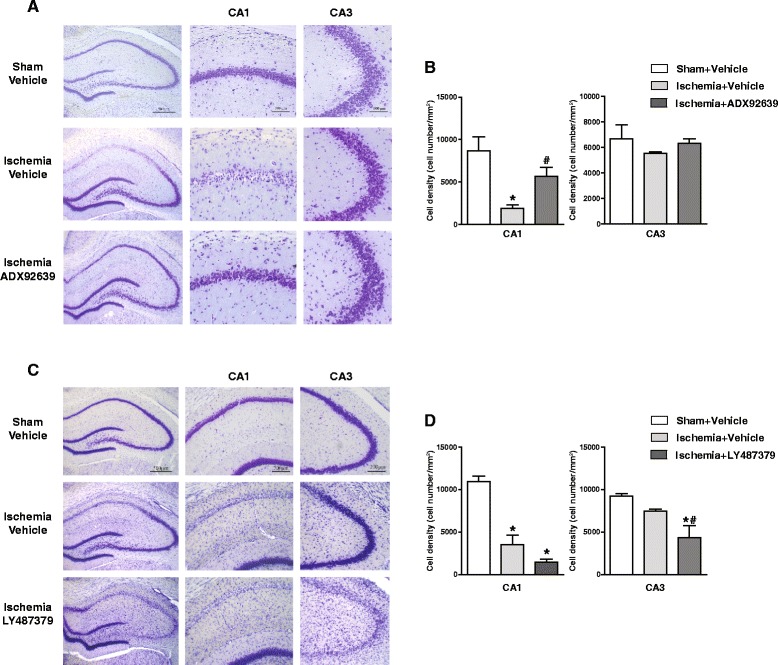
Neuronal damage analysis of post-ischemia treatment with an mGlu2 receptor NAM, ADX92639, or PAM, LY487379. Nissl staining and neuronal density in the CA1 and CA3 regions of sham-operated and 4-VO rats treated with selective mGlu2 receptor ligands. In (**a**) and (**b**), vehicle or the mGlu2 receptor NAM, ADX92639 (30 mg/kg), were orally administered three times at 12, 36, and 60 h after reperfusion; sham-operated rats were only treated with vehicle. Rats were killed at 72 h after reperfusion. Values are means ± S.E.M. of 3-8 rats per group. *p* < 0.05 vs. sham-operated rats (*) or vs. 4-VO rats treated with vehicle (#) (One-way ANOVA + Fisher LSD, F_(2,18)_ = 10). In (**c**) and (**d**), vehicle or the mGlu2 receptor PAM, LY487379 (30 mg/kg), were s.c. injected five times at 12, 24, 36, 48 and 60 h after reperfusion. Sham-operated rats were only treated with vehicle (data of two sham-operated rats treated with LY487379 are reported in the Results section). Rats were killed at 72 h after reperfusion. Values are means ± S.E.M. of 3-5 rats per group. *p* < 0.05 vs. sham-operated rats (*) or vs. 4-VO rats treated with vehicle (#) (One-way ANOVA + Fisher LSD; CA1: F_(2,12)_ = 29.7,*p* < 0.05; CA3 : F_(2,12)_ = 5.9, *p* < 0.05)

In a first set of experiments, 4-VO rats were treated orally with 30 mg/kg of ADX92639 or vehicle at 12, 36, and 60 h after reperfusion. Sham-operated rats were only treated with vehicle. CSF exposure of ADX92639 after an oral dose of 30 mg/kg is largely (4 fold) above the *in vitro* IC_50_ value at the mGlu2 receptor; in addition, 30 mg/kg is the dose of ADX92639 that has previously shown to produce maximal effects in several behavioural models of cognition [36]. Treatment with ADX929639 in 4-VO rats was highly protective against ischemic neuronal death in the CA1 region at 72 h after reperfusion. As expected, global ischemia did not cause detectable changes in the number of viable neurons in the CA3 region, regardless of treatment with ADX92639 (Fig. 6a, b).

Additional groups of 4-VO rats were treated s.c. with the mGlu2 PAM, LY487379 (30 mg/kg) or vehicle at 12, 24, 36, 48, and 60 h after reperfusion. Sham-operated rats were also treated with LY487379 or vehicle at the corresponding times. Treatment with LY487379 in 4-VO rats caused a non-significant trend to the amplification of neuronal death in the CA1 region, and, interestingly, extended ischemic damage to the CA3 region (Fig. 6c, d). The two sham-operated rats treated with LY487379 showed no signs of neuronal death in the CA1 region (number of viable neurons = 10,601 and 9599/mm^2^) with respect to sham-operated rats treated with vehicle (see Fig. 6c), suggesting that the compound was not neurotoxic on its own (i.e., in the absence of ischemia).

## Discussion

The Grm2 gene encoding the mGlu2 receptor is highly plastic and undergoes epigenetic modifications in response to environmental stressors and drug treatments. For example, exposure to prenatal stress, activation of mineralcorticoid receptors by corticosterone, and treatment with atypical antipsychotic drugs all cause an epigenetic down-regulation of mGlu2 receptors in forebrain regions, such as the hippocampus and frontal cortex [29, 37, 38]. Expression of mGlu2 receptors is heavily regulated by acetylation mechanisms [38–41], and HDAC2 in particular was found to bind to the Grm2 promoter and inhibit mGlu2 receptor expression [29]. A high sensitivity to epigenetic regulation is a distinct feature of the mGlu2 receptor. Of note, the mGlu3 receptor, which shares structural and functional similarities with the mGlu2 receptor, is less susceptible to epigenetic regulation [29, 37, 40].

Present data offer the first demonstration that a specific insult (transient global ischemia) causes epigenetic changes in mGlu2 receptors that might be related to mechanisms of neurodegeneration/neuroprotection. Expression of mGlu2 receptors in the CA1 region was down-regulated in response to ischemia at times that preceded the anatomical evidence of neuronal death. Histone acetylation at the Grm2 promoter was also reduced in CA1 at early times after reperfusion (12 and 24 h), whereas HDAC2 and its transcriptional activator, GR [27], showed an early up-regulation (12 h) following reperfusion. HDAC2 levels decreased in the CA1 region at 24 h after reperfusion perhaps as a result of a negative feedback regulation. These data suggest the following scenario: transient global ischemia causes the GR-mediated induction of HDAC2, which, in turn, associates with the Grm2 gene promoter causing a long-lasting histone deacetylation and inhibition of gene expression. After global ischemia, the mGlu2 receptor was down-regulated in the vulnerable CA1 region, but not in the resistant CA3 region. This raised two questions that may be relevant to the study of selective neuronal vulnerability to ischemic damage: (i) is the down-regulation of mGlu2 receptors neurotoxic or neuroprotective?; (ii) why is the mGlu2 receptor specifically down-regulated in the CA1 region after ischemia? Studies carried out in neuronal cultures challenged with excitotoxins or β-amyloid peptide suggest that activation of mGlu2 receptors is detrimental to neurons at least within the context of a neurodegenerative process [22, 23]. Present data are fully consistent with this hypothesis. We used a NAM (compound ADX92639), which showed a high degree of selectivity for mGlu2 receptors and did not cause changes in body temperature following systemic administration. ADX92639, given orally several hours after reperfusion, was highly protective against CA1 damage. In contrast, pharmacological enhancement of mGlu2 receptors with the selective PAM, LY487379, increased neuronal damage. This suggests that the epigenetic down-regulation of mGlu2 receptors occurring in the CA1 region at 12-24 h after reperfusion represents a potential defensive mechanism. However, the residual activity of mGlu2 receptors still contributes to the progression of neuronal damage unless the receptor is pharmacologically inhibited in the post-ischemic period.

The specificity of mGlu2 receptor changes for the CA1 region could be simply explained by assuming that it is only this region that is sensitive to ischemic insult. However, global ischemia caused an early up-regulation of the death-related protein, GADD45β, in both CA1 and CA3 (see also ref. [28]). This suggests that a death gene program is induced in both vulnerable and resistant neurons after global ischemia, and that resistant neurons harbor a molecular repertoire that restrains the development of the program. We were surprised to find that mGlu2 receptor mRNA levels were much lower in CA3 than in CA1 both under basal conditions and in response to ischemia. The low expression of mGlu2 receptors might contribute to the intrinsic resistance of CA3 neurons to ischemic damage unless the function of these receptors is pharmacologically amplified at critical time windows after reperfusion. This explains why post-ischemic treatment with the mGlu2 receptor PAM, LY487379, extended neuronal death to the CA3 region, which was otherwise resistant. We wish to highlight that LY487379 did not cause any apparent sign of neuronal toxicity in non-ischemic rats, suggesting that pharmacological activation of mGlu2 receptor is not toxic *per se* but may play a permissive role on neuronal damage in the presence of an ischemic insult.

The molecular mechanism by which activation of mGlu2 receptors becomes detrimental for neurons committed to die is unknown. mGlu2 receptors are coupled to G_i_/G_o_ proteins and their activation leads to inhibition of adenylate cyclase activity and inhibition of voltage-sensitive Ca^2+^ channels with ensuing reduction of neurotransmitter release [42]. It is possible that either inhibition of GABA release from CA1 interneurons [43, 44] or activation of intracellular death-related mechanisms in pyramidal neurons are involved in the permissive role of mGlu2 receptors on neuronal damage. However, our analysis of Grm2 expression was not carried out at cellular level, and we cannot exclude that mGlu2 receptors present in cells other than pyramidal neurons are involved in the pathophysiology of ischemic neuronal death and can be targeted by neuroprotective drugs. For example, inflammatory mechanisms and microglial activation contribute to neuronal degeneration in models of transient global ischemia, including the 4-VO model [45–47]. mGlu2 receptors are present in microglia [48, 49], and activation of mGlu2 receptors induces a pro-inflammatory and neurotoxic phenotype in microglia [48, 50, 51]. Microglial cells respond to mGlu2 receptor activation with an enhanced production of tumor-necrosis factor-α and other mechanisms that are detrimental to neighbor neurons [50, 51]. On the basis of data obtained in an *in* vitro model of oxygen-glucose deprivation, it has been suggested that activation of microglial mGlu2 receptors by the glutamate released from ischemic neurons may contribute to the overall process of neuronal death [51]. Thus, it cannot be excluded that mGlu2 receptor blockade in microglia might have contributed to the protective effect of ADX92639 we have seen in the 4-VO model.

Our findings raise the possibility that mGlu2 receptor NAMs might be helpful in the treatment of cardiac arrest, hypovolemic shock, severe hypotension or other pathologies that may reduce brain perfusion below a critical threshold and cause damage of vulnerable hippocampal neurons. To support this possibility it will be important to extend the study to other mGlu2 receptor NAMs and also to induce transient global ischemia in rats lacking mGlu2 receptors [52, 53]. Of note, preclinical studies show that mGlu2 receptor NAMs have a potential therapeutic value in the treatment of cognitive dysfuntion associated with major depression, which is often resistant to conventional antidepressant medication [54, 55]. The neuroprotective effect of mGlu2 receptor NAMs may be an added value if these drugs are used for the treatment of depression associated with vascular dementia or chronic cardiovascular disorders causing brain hypoperfusion and neuronal damage.

## Conclusions

We found that the mGlu2 receptor was epigenetically down-regulated in the vulnerable CA1 region during the development of ischemic damage. This change might represent a protective mechanism aimed at restraining ischemic neuronal damage, because post-ischemic treatment with an mGlu2 receptor NAM reduced the extent of ischemic damage. These findings may be relevant in the mGlu receptor field for three reasons. First, they suggest that expression of mGlu2 receptors undergoes plastic changes under pathological conditions and this mechanism might be related to the intrinsic vulnerability of particular neuronal populations to ischemic damage. Second, they are in line with previous findings from our group demonstrating that pharmacological activation of mGlu2 receptors, which is safe under normal conditions, may be detrimental to neurons challenged by a toxic insult. Third, and more important, they suggest that selective mGlu2 receptor NAMs may be beneficial in the treatment of brain ischemia or hypoperfusion.

## Materials and methods

### Induction of transient global ischemia in rats

Adult male Sprague Dawley rats (Charles River, Calco, Italy), weighing 200-250 g, were housed under controlled conditions (ambient temperature, 22 °C; humidity, 40 %) on a 12 h light-dark cycle with food and water *ad libitum*. Animal experiments were performed in full compliance with the ARRIVE guidelines. All efforts were made to minimize the number of animals and animal suffering. The experimental protocol was approved by the Ethical Comittee of Neuromed Institute (Pozzilli, Italy) and by The Italian Ministery of Health (D.M. 227/2011-B). Transient global ischemia was induced by the 4-VO method [3], as described previously [5] with minor modifications. In brief, animals were anesthetized with chloral hydrate (350 mg/kg, i.p.), and positioned in a stereotaxic apparatus with the head tilted down at approximately 30° from the horizontal plane; an incision was made behind the occipital bone directly overlying the first two cervical vertebrae. The paraspinal muscles were separated from the midline, and the right and the left alar foramina of the first cervical vertebrae were exposed. A 0.5 mm electrocautery needle was inserted through each alar foramen, and both vertebral arteries were electrocauterized and permanently occluded. Afterwards, both common carotid arteries were exposed and isolated with a 3-0 silk tread, and the incision was sutured. Twenty-four hours later, animals were anesthetized with isofluorane (induction, 3 %; maintenance, 2 %), the wound was reopened, and both common carotid arteries were occluded with aneurismic clips for 10 min. Anesthesia was temporarily discontinued during carotid artery occulsion. Only rats showing complete loss of consciousness with loss of righting reflex and bilateral midriasis were included in the study. After 10 min of occlusion, isoflurane anesthesia was restored, both carotid arteries were reopened and visually inspected to ensure adequate reflow, and the wound was sutured. Body temperature was monitored and manteined at 37 °C with rectal thermistor coupled to a heating blanket. After reperfusion, animals were kept for 2 h at 37 °C before being treansferred to their home cages. For sham operation, animals were subjected to the same anesthesia and surgical procedures, except that carotid arteries were not occluded.

### Biochemical analysis

Animals were killed by decapitation at 6, 12, 24, and 72 h after reperfusion. The brain was quickly removed and placed on an ice-cold rat brain matrix. A 2-mm thick slice containing the dorsal hippocampus was rapidly removed, and the CA1 and CA3 subfileds were microdissected on ice under stereomicroscope, immediately frozen on liquid nitrogen, and stored at -80 °C.

### RNA isolation, reverse transcription and qPCR

Total RNA from CA1 and CA3 hippocampal regions was extracted using Trizol reagent (Invitrogen) according to manufacturer’s protocol. The RNA was then treated with DNAse (Qiagen) and single strand cDNA was synthesized from 2 μg of total RNA using superscript III (Invitrogen) and random hexamers. Real-time PCR was performed on 20 ng of cDNA by using specific primers and Power SYBR Green Master Mix (Applied Biosystem) on an Applied Biosystems Step-One instrument. Thermal cycler conditions were as follows: 10 min at 95 °C, 40 cycles of denaturation (15 sec at 95 °C), and combined annealing/extension (1 min at 60 °C). Primers used were as follows: Hdac1 *Forw* CTGTCCGGTATTTGATGGCT and *Rev* TCAGACTTCTTCGCATGGTG; Hdac2 *Forw* AGGTCGTAGGAATGTCGCTG and *Rev* GATTTGGCTCCTTTGGTGTC; Hdac3 *Forw* CCAGAAGCACCCAATGAATT and *Rev* TTCCAAACCCGTTACCAGAG; mGlu1 receptor *Forw* CATACGGAAAGGAGAAGTGA and *Rev* AAAAGGCGATGGCTATGATA; mGlu5 receptor *Forw* GCTGTGAGATCAGAGATTCCTGC and *Rev* ACTCCCACTATGGGTTTCTTGG; mGlu2 receptor *Forw* GCTGCTCCAAGGATACAC and *Rev* ATAGCTGATCTGTGGGATCT; mGlu3 receptor *Forw* CAAGTGACTACAGAGTGCAG and *Rev* CTGTCACCAATGCTCAGCTC; Dnmt1 *Forw* TGTCCTGTCGTCTGCAACCT and *Rev* GCCATCTCTTTCCAAGTCTTT; Dnmt3a *Forw* CCATGCCAAGACTCACCTTC and *Rev* GCTTTCTTCTCAGCCTCCCT; Nr3c1 *Forw* CCATCGTCAAAAGGGAAGGG and *Rev* CAGCTAACATCTCTGGGAAT; Gadd45β *Forw* GCTGGCCATAGACGAAGAAG and *Rev* GCCTGATACCCTGACGATGT; and β-actin *Forw* GTTGACATCCGTAAAGACC and *Rev* TGGAAGGTGGACAGTGAG.

mRNA copy number of each gene analyzed was calculated from serially diluted standard curves simultaneously amplified with the samples and normalized against β-actin copy number.

### Chromatin immunoprecipitation

Chromatin immunoprecipitation assay was performed by standard procedures. Briefly, tissue was cross-linked using 1 % formaldehyde at room temperature for 10 min. The crosslinking reaction was stopped by adding glycine to a final concentration of 0.125 M. Tissue was then washed three times with ice-cold PBS supplemented with protease inhibitors (Sigma-Aldrich), homogenized in lysis buffer (5 mM PIPES pH 8.0, 85 mM KCl, 0.5 % NP40, protease inhibitors), and nuclei lysis buffer (1 % SDS, 10 mM EDTA, 50 mM Tris-HCl pH 8.0, protease inhibitors), and then sonicated on ice using a Covaris S220 ultrasonicator. Following sonication, chromatin fragments of 0.2-0.6 kb in length were obtained. Ten percent of the sonicated lysate was saved and successively used to quantify the total amount of DNA present in different samples before immunoprecipitation (Inputs). The chromatin solution was precleared with salmon sperm DNA/protein A-agarose 50 % gel slurry for 1 h at 4 °C and immunoprecipitated overnight at 4 °C with antibodies against acetyl-histone H3 (Upstate) or the respective isotype matched control Ig. After precipitation, the chromatin-antibody complexes were collected using Protein A Sepharose beads and washed. Samples were then eluted with 1 % SDS, 100 mM NaHCO_3_ at room temperature for 15 min, reverse cross-linked with NaCl 100 mM at 65 °C overnight, and treated with proteinase-K. Protein-free DNA was extracted by phenol/chloroform/isoamyl alcohol, precipitated with 100 % ethanol, and analyzed by real-time PCR using the following specific primers for Grm2 promoter: *Forw* GATCTGCTGGAAGCTGCTG and *Rev* CCTCCTCTGTTCCTCTGGACT. Levels of histone acetylation at the mGlu2 receptor gene promoter were expressed as percentage of the input DNA that was immunoprecipitated by the anti acetyl-histone H3 antibody using the following equation: % (acetDNA ‐ IP/total input) = 2 ^ [(Ct(10 % input) ‐ 3.32) ‐ Ct(acetDNA ‐ IP)] × 100 %.

### Western blot analysis

CA1 regions were dissected and homogenized at 4 °C in a buffered solution composed of Tris-HCl pH 7.5, 10 mM; NaCl, 150 mM; SDS 10 %, EDTA, 5 mM; PMSF, 10 mM; IGEPAL, 1 %; leupeptin, 1 μg/ml; and aprotinin, 1 μg/ml. Equal amounts of proteins (30 μg) from supernatants were separated by 10 % SDS polyacrilamide. After separation, proteins were transferred on immuno-blot PVDF membranes. Membranes were incubated with a mouse monoclonal anti-HDAC2 antibody (1:5,000, overnight at 4 °C, Upstate), and then incubated for 1 h with an anti-mouse secondary antibody (1:7000, peroxidase-coupled). Immunostaining was revealed by the enhanced ECL Western blotting analysis system (Hybond ECL, GE Healthcare Europe). The blots were reprobed with anti-β-actin monoclonal antibody (1:60,000; Sigma) followed by an anti-mouse antibody (1:7,000; Calbiochem). Densitometric analysis was performed with Quantity One software (Bio-Rad).

### Histological analysis

Animals were killed at 24 or 72 h after reperfusion. Brains were fixed in Carnoy’s solution, embedded in paraffin, and sectioned at 10 μm. Sections were deparaffinized and processed for staining with thionin (Nissl staining) for histologic assessment of neuronal degeneration. Surviving neurons were counted in the CA1 and CA3 hippocampal subfield under a light microscope at 20× magnification. Neurons with a rounded shape similar to that commonly observed in sections from control animals were considered to be viable. Cells were counted from three sections of the dorsal hippocampus within a dissector area of 1,225 μm^2^ (35 × 35 μm) randomly positioned by the software (Image pro plus 6.0) over the region of interest. The results are expressed as cell density per mm^2^.

### Drugs

2,2,2-trifluoro-N-[4-(2-methoxyphenoxy)phenyl]-N-(3-pyridinylmethyl)ethanesulfonamide hydrochloride (LY487379) was purchased from Tocris. ADX92639, was provided from Addex Therapeutics. The molecular structure of ADX92639 could not be disclosed by Addex. General features of compound ADX92639 are the following: molecular weight = 332; partition coefficient (ClogP) = 1.8; topographical polar surface area (tPSA) = 79 Ȧ; H-bond donor/H-bond acceptor (HBD/HBA) = 0/6; kinetic solubility at pH 7.4 = 0.137 mg/ml; intrinsic clearance (Clint) in rats = 12 μl/min/mg prot.

### Characterization of the receptor profile of ADX92639

#### FLIPR-based intracellular Ca^2+^ mobilization assay

A fluorescent cell-based Ca^2+^ mobilization assay was performed on HEK293 cells stably expressing rat or human recombinant mGlu receptor subtypes, as described previously [56]. ADX92639 was tested at different concentrations, up to 30 μM, as an agonist, positive allosteric modulator (PAM), or negative allosteric modulator (NAM). ADX92639 was also tested in competition binding assay on membranes expressing 71 targets including G-protein coupled receptors, membrane transporters, enzymes, and ion channels (Cerep, Poitiers, France). In a Schild-plot analysis in mGlu2 receptor-expressing cells, the concentration-response curve to glutamate was tested in the presence of increasing concentrations of ADX92639. To assess the reversibility of the effect of ADX92639, cells expressing the mGlu2 receptor were treated with the compound and then either washed three times with PBS or not. Cells were then stimulated with glutamate at the EC_80_ value.

### [^3^H]LY341495 binding on membranes from cells expressing human mGlu2 receptor

For the study of [^3^H]LY341495 binding, we used the same buffer and conditions described by Wright et al. (2001). Cell membrane homogenates were washed three times with ice-cold assay buffer (10 mM potassium phosphate buffer containing 100 mM potassium bromide, pH 7.6). Potassium bromide was added because bromide ions are known to enhance [^3^H]LY341495 binding [57]. Membranes were incubated in the presence of 1 nM [^3^H]LY341495 at 5 °C for 30 min in the presence of increasing concentrations of non-radioactive LY341495, glutamate, or ADX92639. Bound and free [^3^H]LY341495 were separated by filtration.

### Measurements of body temperature in rats treated with ADX92639

The effect of ADX92639 on body temperature was measured in male Sprague-Dawley rats of the same age and weight of those used for the induction of global ischemia. Rats were treated orally with either ADX92639 (30 mg/kg, suspended in 1 % carboxymethylcellulose) or vehicle. ADX92639 and vehicle were administered three times at time 0, and then after 24 and 48 h. The rectal temperature was measured at different times after the first administration of either ADX92639 or vehicle (see Table 1) by a thermistor inserted about 2 cm into the rectum. The probe was left in place for about 10 sec.

### Drug treatments in 4-VO rats

Twenty-six 4-VO rats satisfying the inclusion criteria (loss or righting reflex, no response to painful stimuli, and midriasis during carotid artery occlusion), and 8 sham operated rats were used for pharmacological experiments. Two groups of 4-VO rats (*n* = 8 per group) received oral administration of ADX92639 (30 mg/kg, suspended in 1 % carboxymethylcellulose) or vehicle. ADX92639 or vehicle were administered three times in the post-ischemic period at 12, 36 and 60 h after reperfusion. Three sham-operated rats receving three oral administration of 1 % carboxymethylcellulose at the same time-points were used as controls. No sham operated rats received ADX92639. Two additional groups of 4-VO rats (*n* = 5 per group) were injected s.c. with either LY487379 (30 mg/kg, dissolved in arachid oil) or vehicle at 12, 24, 36, 48 and 60 h after reperfusion. Sham-operated rats were treated s.c. with either LY487379 (*n* = 2) or vehicle (*n* = 3) at the same time-points. All animals were killed at 72 h after reperfusion for histological assessment of neuronal death in the CA1 and CA3 regions.

## Abbreviations

4-VO: 4-vessel occlusion
PAM: positive allosteric modulator
NAM: negative allosteric modulator
